# Characterization of a novel scale maille contralateral breast shield: SMART Armor

**DOI:** 10.1002/acm2.12158

**Published:** 2017-08-11

**Authors:** Macinley Butson, Susan Carroll, Martin Butson, Robin Hill

**Affiliations:** ^1^ The Illawarra Grammar School Mangerton NSW Australia; ^2^ Department of Radiation Oncology Chris O'Brien Lifehouse Camperdown Australia; ^3^ Institute of Medical Physics University of Sydney Camperdown Australia

**Keywords:** breast, contralateral breast, radiation shielding, radiotherapy, skin dose

## Abstract

During breast radiotherapy treatment, the contralateral breast receives radiation doses to the skin and subcutaneous tissue caused mainly from incident electron contamination and low energy photon scatter radiation. Measurements have shown that for a typical hybrid tangential treatment, these dose levels can be up to 17% of maximum applied prescription dose if no shielding is used during the treatment process. This work examined the use of different shielding metals, aluminum, copper, and lead to reduce peripheral radiation dose to evaluate the optimal metal to form the basis of a contralateral breast radiation shield. This work also shows a simple but novel method to substantially reduce this unwanted radiation dose with the use of a copper scale maille sheet which can be easily and accurately draped over a patient's contralateral breast during treatment. The copper scale maille is flexible and can thus conform around typical breast shapes. It can also form irregular shaped edges to match those outlined by typical tangential treatment fields. As the shield is made from copper, it is nontoxic and can potentially be used directly on patients for treatment. The designed copper scale maille has shown to reduce contralateral breast skin and subcutaneous dose by up to 80% for typical radiation fields used in breast radiotherapy.

## 1 INTRODUCTION

One in eight women will develop breast cancer in their lifetime and it is the most common cancer in women.^1^ It is recommended that radiotherapy treatment is delivered after initial surgery for breast cancer to substantially reduce the risk of site specific relapse. However, during breast cancer treatment using radiotherapy, the other breast (the contralateral breast) receives radiation dose as an unwanted side effect of the treatment. The association between low dose from peripheral ionizing radiation and the risk for secondary cancer has attracted interest specifically for the long‐term surviving patients.^2^, ^3^, ^4^, ^5^, ^6^ Specifically, concerns regarding oncogenesis and second cancer induction are realized and invoke the need for ALARA (As Low As Reasonably Achievable) principles to be followed.

During radiation therapy, regardless of the treatment technique, the surrounding normal tissue outside the treated area inevitably receives some amount of radiation dose. Such dose outside the geometric boundaries of the treatment fields is known as peripheral dose. There are three main sources of peripheral dose: (a) leakage through the treatment collimation (x‐rays); (b) scattered radiation from the secondary collimators and beam modifiers such as the MLC, physical wedges (x‐rays and electrons); and (c) internal scatter originating in the patient (x‐rays).^7^, ^8^ Butson et al. 9 showed that peripheral doses can be as large as 20% of maximum dose for normally incident beams and that these values can increase with oblique angle of incidence.^10^

To minimize radiation doses delivered to the contralateral breast, lung, and heart, some patients can be treated with a prone technique.^11^ If a supine treatment is used, to reduce contralateral breast dose, different types of shielding devices, and delivery techniques have been used.^12^, ^13^, ^14^, ^15^, ^16^ These included mobile high‐density lead shields placed between the treatment machine and the patient. Other devices used were tissue‐density superflab material laid over the patient's contralateral breast. Although these methods did reduce contralateral breast dose, they presented technical difficulties in their usage. Mobile lead shields need to be placed appropriately between the patient and the treatment head of the linac. Such techniques are not very efficient since they demand precise positioning alignments. They also suffer from not being able to be shaped around the treatment field edges. Superflab bolus can also reduce skin and subcutaneous dose but it requires at least 10 mm thickness of bolus material to provide sufficient attenuation. This process may also introduce misalignment errors near the edge of the treatment fields. Interestingly, materials like brash mesh (chain mail) can be used to increase skin dose on the treated breast for some patients when required.^17^

In this study, we evaluate shielding characteristics of a novel copper scale maille sheet for potential use in contralateral breast dose reduction.

## 2 MATERIALS AND METHODS

Shielding properties of various metals were studied in the peripheral region of 6 MV x‐ray beams produced by a Varian 6EX linear accelerator (Varian Medical Systems, Palo Alto, CA, USA). The materials evaluated were 1.0 mm thick aluminum, 1.0 mm copper, and 1.0 mm lead sheets. Dose measurements were performed in RMI solid water (RMI, Middleton, WI, USA) using an Attix model 449 parallel plate ionization chamber (RMI, Middleton WI, USA) at depths of 1 mm, 2 mm, 3 mm, 5 mm, 10 mm, and 15 mm. Measurements were made 5 cm away from the edge of the primary field which was a 10 cm × 20 cm field size at 100 cm source to surface distance (SSD). Results were compared to measured percentage dose at the same peripheral position for an open field with no metal shielding in place. The results were normalized to 100% at the depth of maximum dose at the central axis of the primary radiation field (depth of 15 mm). The measurements were repeated 6 times for uncertainty analysis. Errors were calculated as 2 standard deviations of the mean for all measurements taken at each measurement point. These errors combine both type A and type B errors associated with uncertainty in set up as well as deviations in measurement accuracy. Errors are expressed as the square root of the sum of the squares of each error in relation to measurements made and is expressed by eq. (1).

(1) $$\delta R=\surd \{{\left(\delta x\right)}^{2}+{\left(\delta y\right)}^{2}+{\left(\delta z\right)}^{2}\}$$

where *δ*R is the total error, and *δ*x, *δ*y, and *δ*z represent each component of measured uncertainty.

**Figure 1:**
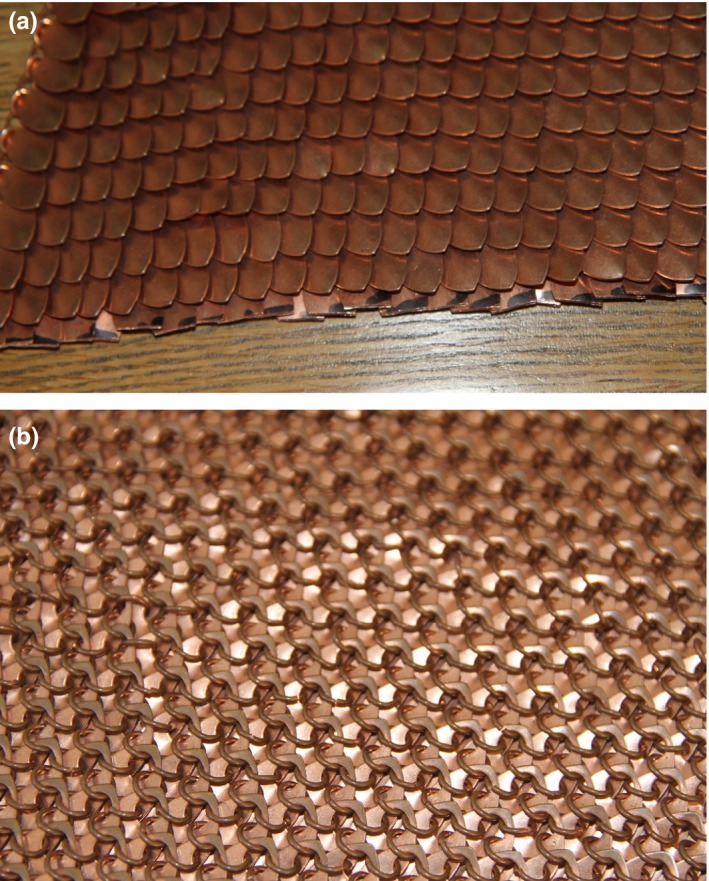
(a) SMART Armor peripheral dose shield. Front View. (b) SMART Armor peripheral dose shield. Rear View.

Dose measurements were also made on the shielding characteristics of a scale maille designed peripheral dose shield. The SMART Armor (Scale Maille Armor for Radiation Therapy) was made from 12 mm × 22 mm × 0.6 mm thick copper scales, interwoven together to form a scale maille design as shown in Fig. 1. Conventional scale maille weaving techniques were employed to create the scale maille. This utilizes the use of 7 mm diameter jumper rings linked together and the 0.6 mm thick copper scales threaded over the jumper rings through a 2 mm diameter hole located at the top of each copper scale. By interweaving the scale maille pattern, the 0.6 mm thick copper scales overlap producing a 1.2 mm thick copper shield at all points. The underside of the scale maille is shown in Fig 1b.

The scale maille SMART Armor sheet has dimensions of 30 cm × 30 cm × 0.3 cm thick. This is ample size to cover a typical contralateral breast. To utilize the SMART Armor, the sheet can be easily draped over the contralateral breast region during treatment, ensuring that the shield does not interfere with any entry fields. This would cause increases in skin dose due to build up dose effects. The shield conforms to the breast shape and provides protection during treatment. The shield would not need to be present during simulation or CT as it does not affect treatment dose and treatment should not occur through the device.

The design of the shield allows the copper scales to overlap thus providing an approximate 1.2 mm thickness of copper over the entire region of the shield. The design allows the SMART Armor to conform to the shape of the contralateral breast providing substantial coverage and shielding. The shield edge of the SMART Armor can be shaped to follow the irregular field edges produced by typical cancer treatments for radiotherapy. The Smart Armor can be handled safely as it is made from copper, and is thus nontoxic and would be easy for radiation therapy workers to use on patients.

**Figure 2:**
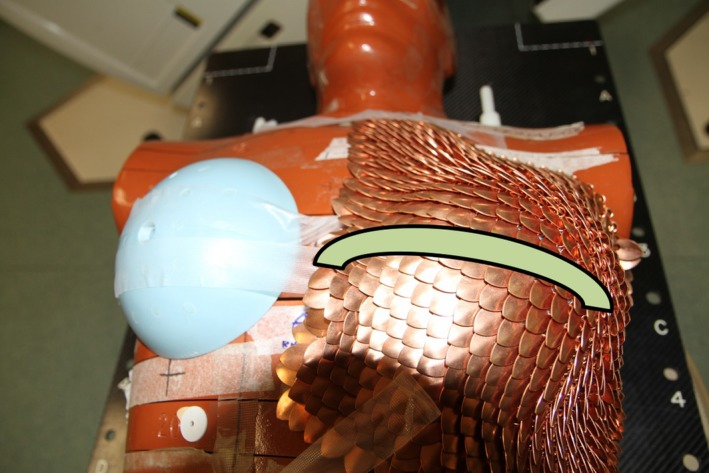
ART anthropomorphic phantom layout with the SMART Armor shield in place.

To evaluate contralateral breast shielding, an ART anthropomorphic phantom, as shown in Fig. 2, was positioned on a Varian 21EX linear accelerator and treated with a conventional 10 cm × 20 cm asymmetric parallel opposed field size using a medial and tangent beam configuration with 6 MV x‐ray beams. Skin doses were measured using a Gafchromic EBT3 film (Ashland Inc, New Jersey, USA) from 5 cm inside the medial edge of the medial beam and across the contralateral breast. Gafchromic films have been shown to be suitable for accurate skin dosimetry.^18^, ^19^ Again, the measurements were repeated six times for reproducibility and uncertainty analysis. The doses were normalized to 100% delivered dose at the midpoint position in the treated breast. The skin dose results were compared to percentage dose results delivered without the SMART Armor in position. The measured dose represented the sum of radiation dose delivered from both the medial and lateral beams. To evaluate SMART Armor using different types of clinical treatments, five clinical plans from different patient treatments were delivered to the ART phantom and skin dose assessed with and without the SMART Armor shield.

The five patient treatments delivered included, patient one, using enhanced dynamic wedge fields, patients two and three, using field in field techniques, and patients four and five, using a hybrid intensity modulated radiation therapy (IMRT) technique. Physical wedges were not used for patient treatments and thus were not evaluated. Results for skin dose were measured using the same techniques as the open field measurements. To perform the irradiations, the patient plans were transferred to the ART phantom CT dataset for planning and treatment delivery. It is acknowledged that the plans would not be optimized due to differences in anatomy; however, this work would highlight differences in contralateral breast skin dose delivered, with and without the SMART Armor shields.

## 3 RESULTS

**Figure 3:**
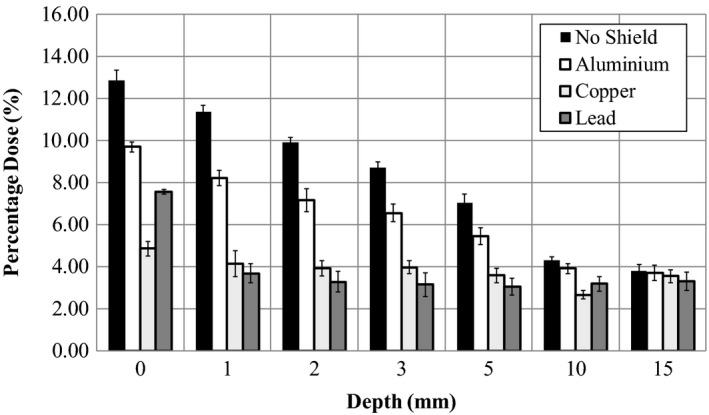
Percentage of D_max_ doses delivered to the phantom with various shielding materials in the peripheral region.

Figure 3 shows the results measured for attenuation of the radiation beam when the different metals are used to attenuate radiation in the beams peripheral region and compared to no shield results. Results were measured at depths ranging from 0 mm (at the skin surface) down to 15 mm, well beyond the subcutaneous tissue region. As can be seen in this configuration, at the surface when no shield was in place, approximately 13% of maximum dose was delivered. This was reduced to 9.5% for aluminum, 4.5% for copper and 7.2% for lead. A comparison of these values in dose reductions for three metals is shown in Table 1. For example, at 5 mm depth, the aluminum provides a 22.5% reduction in dose, whereas the copper and lead achieve 49% and 56.7% reductions, respectively.

**Table 1:** Peripheral skin dose reduction with metal shields. Dose reduction achievable with various metals

Depth (mm)	Aluminum	Copper	Lead
0	24.6 ± 4.2	62.2 ± 4.6	41.2 ± 3.8
1	27.6 ± 3.7	63.6 ± 5.3	67.6 ± 4.3
2	27.5 ± 4.7	60.3 ± 3.5	66.8 ± 4.4
3	24.6 ± 3.9	54.3 ± 3.3	63.9 ± 5.0
5	22.5 ± 4.5	49.0 ± 4.2	56.7 ± 4.4
10	9.4 ± 2.2	38.1 ± 2.1	26.3 ± 3.0
15	2.6 ± 3.6	6.6 ± 3.3	13.2 ± 4.2

**Figure 4:**
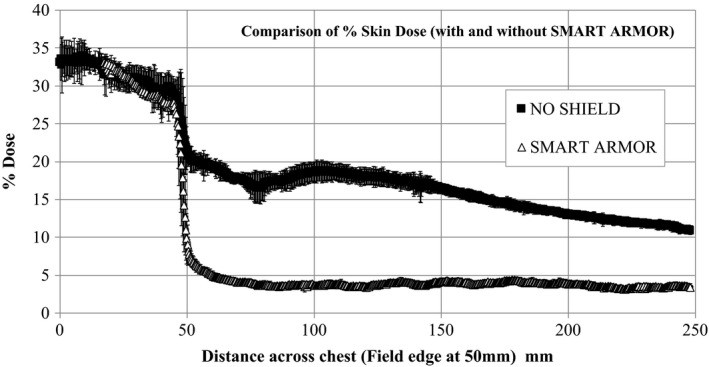
Dose reductions achievable on the contralateral breast with the SMART Armor shield in place.

Figure 4 shows a dose profile measured across the chest wall of the anthropomorphic phantom, with and without the SMART Armor shield in place. The results are measured at an equivalent depth of 0.125 mm which is the effective point of measurement of EBT3 film. The results are normalized to 100% at the midpoint in the treated breast. In this example, the skin dose within the treatment field is similar with and without the shield being approximately 30–35% of maximum. However, in the peripheral region (from 50 mm distance onwards), the skin dose has been substantially reduced by the presence of the SMART Armor being reduced from as high as 16% down to approximately 4%. This represents an up to 75% reduction in dose achievable in the contralateral breast region with the use of the SMART Armor.

**Figure 5:**
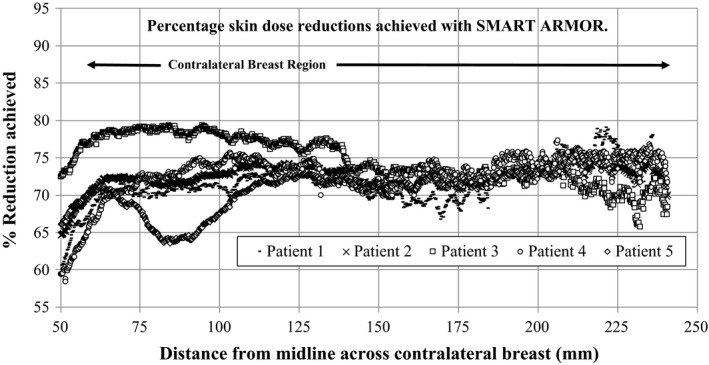
Percentage dose reductions achievable with SMART Armor for enhanced dynamic wedge, field in field and hybrid IMRT delivery techniques.

Figure 5 shows the results for percentage dose reductions achievable across the chest wall of the anthropomorphic phantom. As can be seen, variations in contralateral breast dose with and without the SMART Armor range from approximately 60% to 80% in all five cases studied. In all cases, substantial reductions in skin dose are measured whether the treatment technique utilized enhanced dynamic wedges, field in field techniques, or hybrid IMRT dose delivery.

## 4 DISCUSSION

Dose delivered to the peripheral skin and subcutaneous regions during clinical radiotherapy is mainly caused by incident electron contamination from the entry beams. This contamination originates from production in the air column and the linear accelerator head.^20^ As such, substantial attenuation of this dose can be achieved by peripheral shielding using high‐density materials. Results from Fig. 3 highlight the dose reduction achievable. Of interest is the significant reductions achieved with 1.0 mm of copper which reduced dose levels to below 5% at all depths. This value decreased to just below 4% by 15 mm depth and the majority of the dose remaining at all depths is expected to be from internal radiation scatter and high‐energy x‐ray penetration which was capable of transmission through the linear accelerator tungsten jaws. As the reductions in dose were achieved by removal of electron contamination, dose from posterior beams will not negligibly reduced for the contralateral breast. As such, 1.0 mm of copper material could be considered a useful shielding thickness if dose to peripheral regions were required to be reduced. This is the case for the contralateral breast during breast cancer treatment. Interestingly, lead showed a unique and reproducibly higher dose level directly under its surface compared to copper producing an average 7.6% dose compared to 4.8%. At every other depth beyond the surface, the peripheral measured dose was less for lead than for copper. Our assumptions are that the lead is producing a small quantity of low energy radiation on the exit side which deposits a larger degree of dose at the phantom surface. This does not occur for copper. Aluminum has a much lower density than copper or lead, and thus provides less radiation shielding properties at all depths. As the skin is a radiation sensitive organ, these findings make copper a better suited radiation shield than lead for peripheral regions when 6 MV x‐rays are used for radiotherapy treatment.

As copper is a strong but malleable material it also lends itself well to be used to construct flexible and maneuverable shielding using a scale maille design. The scale maille SMART Armor can conform to the shape of the contralateral breast phantom, cover the irregular shaped treatment field edge as well as providing substantial reductions in delivered peripheral dose. As copper is a nontoxic material and lasts a long time without perishing and/or oxidation, it is well suited for clinical use when a shield is required for reducing the contralateral breast dose. Reductions of up to 80% from original values were achieved with the SMART Armor shield for standard open field tangential treatments. When standard clinical treatments were evaluated including enhanced dynamic wedges, field in field and hybrid IMRT techniques, the dose reductions achieved using SMART Armor remained high. In the five cases studied, the values for percentage dose reduction ranged from 60% up to 80% across the contralateral breast region. As such, SMART Armor can provide substantial contralateral breast shielding during common supine breast irradiation techniques. It should be noted that SMART Armor is only useful during supine breast treatments. Prone techniques are used to recue both contralateral breast and lung dose for certain patients. The SMART Armor when used is only draped over the contralateral breast region and is not placed within the primary breast treatment field. No distinguishable change in primary breast dose was measured or expected with the use of the SMART Armor.

SMART Armor due to its weaved design is easy to use clinically and takes approximately 30 s to align on the anthropomorphic phantom. Clinically this may take longer; however, the authors believe the small increase in time for set up is warranted due to the substantial reductions in contralateral breast dose achieved.

## 5 CONCLUSIONS

High‐density materials, such as copper, can provide substantial shielding effects in radiotherapy cancer treatment in the peripheral regions of megavoltage x‐ray beams. Copper was shown to be superior to lead as a choice of shielding material due to its ability to reduce skin dose to a lower level. Copper was also found to be a useful choice of material to create a scale maille style SMART Armor which can be used to provide protection to skin and subcutaneous tissue in peripheral regions during radiotherapy treatment. This is especially useful in treatment of breast cancer where dose to the contralateral breast can be reduced by up to 80% of original values.

## CONFLICT OF INTEREST

The authors declare no conflict of interest.
